# A tribute to Doris Rosenthal, “La grande Dame de la thyroidologie brésilienne” (1932-2026)

**DOI:** 10.20945/2359-4292-2026-0095

**Published:** 2026-08-23

**Authors:** Denise P. de Carvalho, Rui M. B. Maciel

**Affiliations:** 1 Laboratório de Fisiologia Endócrina Doris Rosenthal, Instituto de Biofísica Carlos Chagas Filho, Universidade Federal do Rio de Janeiro, Rio de Janeiro, RJ, Brasil; 2 Laboratório de Endocrinologia Molecular e Translacional, Disciplina de Endocrinologia, Departamento de Medicina, Escola Paulista de Medicina, Universidade Federal de São Paulo, São Paulo, SP, Brasil

**Figure 1.**
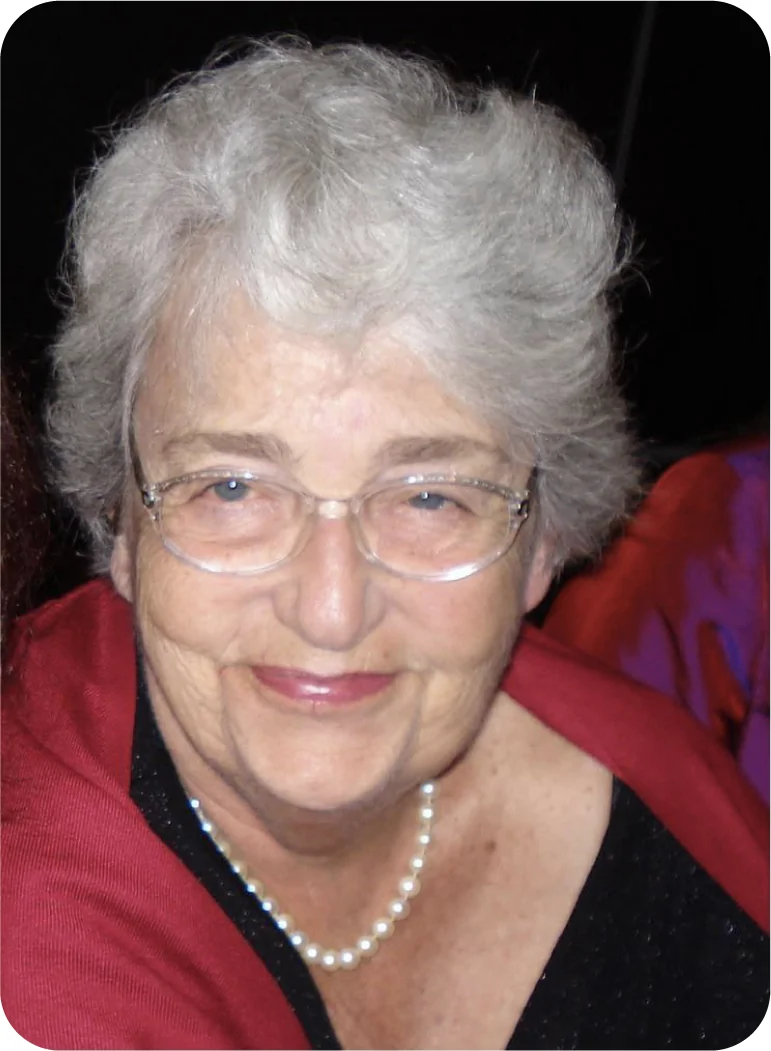
Doris Rosenthal at the American Thyroid Association Annual Meeting, New York, 2007.

Doris Rosenthal (**Figure 1**) passed away on June 5, 2026. With her passing, Brazil lost not only a distinguished scientist and educator, but also a pioneering woman whose life was inseparably intertwined with the advancement of medical education, endocrinology, and scientific research in the country. She also forged several leaders in the field.

**Figure 2.**
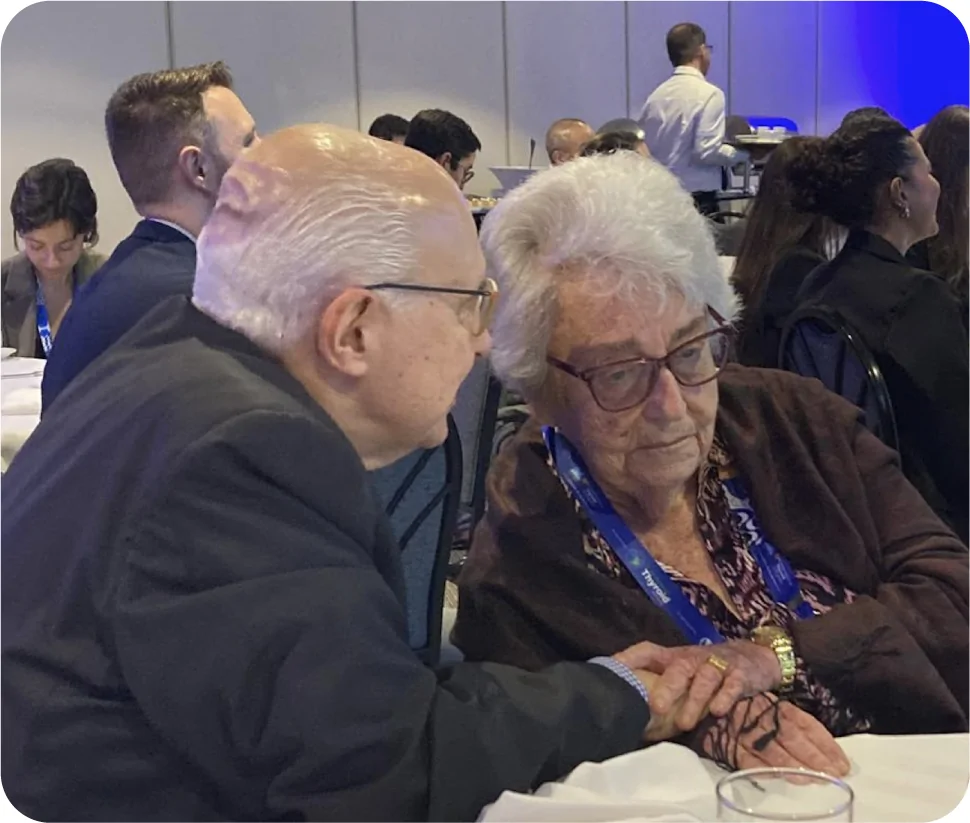
Doris Rosenthal and Rui Maciel at the International Thyroid Meeting in Rio de Janeiro, 2025.

**Figure 3.**
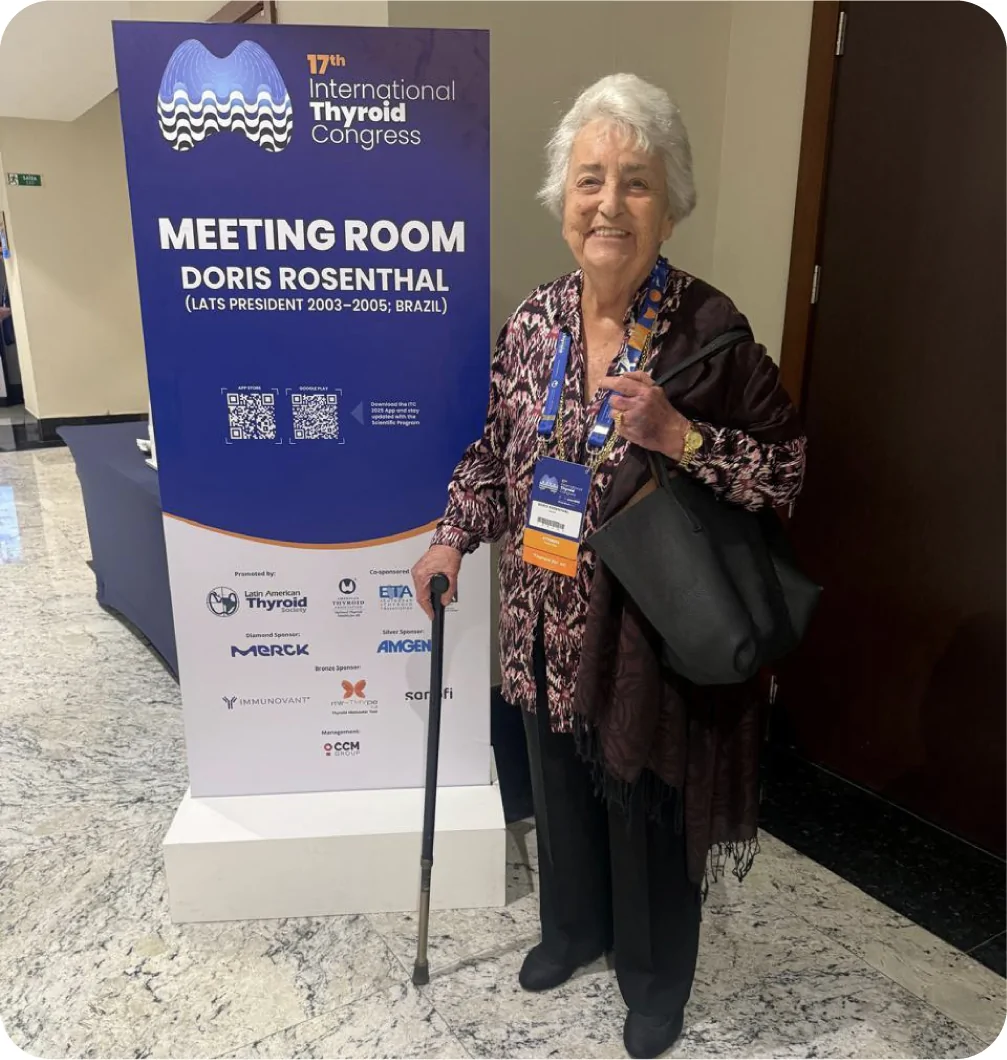
In front of the Meeting room, Doris Rosenthal at the International Thyroid Meeting in Rio de Janeiro, 2025.

**Figure 4.**
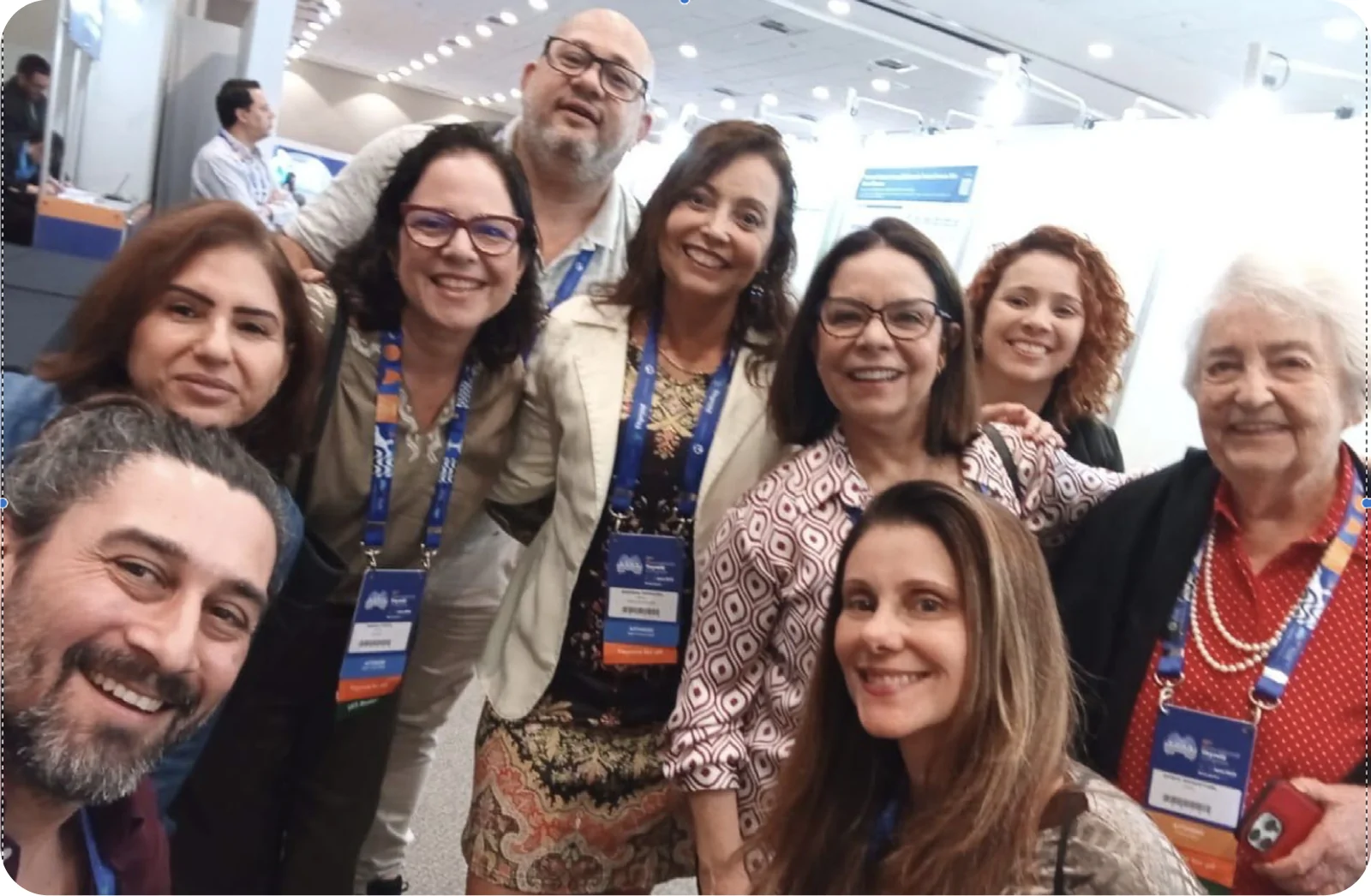
Doris and former students from UFRJ during the International Thyroid Meeting, 2025.

Her presence at scientific conferences, where she would enter the lecture halls eager to follow the latest debates in the field, was always a source of admiration. At the age of 93, at the last International Thyroid Congress, she remained active and continued to inspire those around her (**Figures 2-4**).

Born in Düsseldorf, Germany, on August 23, 1932, Doris experienced the devastating consequences of Nazi persecution in her childhood. The daughter of a Jewish father, Frederick, and a Catholic mother, Maria, she arrived in Brazil with her family in late 1941, where they settled in Rio de Janeiro. Their journey was never easy, and they undoubtedly faced prejudice and hardship. Yet Doris and her family met those challenges with resilience, courage, and dignity, shaping a woman whose unwavering pursuit of knowledge and extraordinary determination became the hallmarks of her life. Her story proves that the struggle for a life of purpose, learning, and humanity was profoundly worthwhile.

Doris earned her medical degree from the State University of Rio de Janeiro (UERJ) in 1957. At the time, few women entered higher education, and even fewer pursued careers in medicine. Brazilian universities had been formally opened to women only in 1879, when Emperor Dom Pedro II issued a decree—seventy-one years after the founding of the Rio de Janeiro School of Medicine—allowing them to enroll.

Until then, women’s education in Brazil had largely been confined to schools that emphasized social etiquette and proper manners. The pioneering women who entered medicine faced restrictions, prejudice, and countless obstacles from the outset of their careers, and only a small number succeeded in establishing themselves in this traditionally male domain. Doris was undoubtedly one of those pioneers. According to Brazil’s 2023 medical demographic data, women were expected to become the majority of physicians in the country by 2024—a prediction that has since become a reality (^1^). Doris helped pave the way for that transformation.

She began her career as a research fellow in 1961. Between 1966 and 1968, she joined the pioneering group of professors at the University of Brasília’s Faculty of Medical Sciences. From 1970 onward, she served as Professor of Endocrine Physiology at the Carlos Chagas Filho Institute of Biophysics at the *Universidade Federal do Rio de Janeiro* (UFRJ), which remained her academic home for more than five decades. Doris also devoted part of her professional life to the *Instituto Estadual de Diabetes e Endocrinologia Luiz Capriglione* (IEDE), where she played a key role in establishing translational thyroid research.

In 1975, she earned her doctoral degree in science from UFRJ’s Postgraduate Program in Biophysics. In 1978, she completed a postdoctoral fellowship at INSERM in France.

At the time, postgraduate education in Brazil was still taking shape, and Doris was among the faculty who played an active role in building what would become one of the country’s great educational achievements. A central element of this success was the integration of undergraduate teaching, graduate education, and scientific research—a model that took root in Brazil in the 1970s. Throughout her career, she developed biochemical assays to measure thyroid metabolites and investigated defects in thyroid hormone synthesis by chromatographing iodinated amino acids. She also played a significant role in studies of iodine deficiency and endemic goiter in Brazil, participating in projects sponsored by the World Health Organization. In 1975, her work on the dynamics of radioactive iodine uptake in thyroid adenomas earned her the affectionate nickname “Madame Hot Nodules”. The nickname remained with her throughout her career and became a symbol of her pioneering contribution to the study of thyroid disease.

As a professor and researcher at the Carlos Chagas Filho Institute of Biophysics at the Federal University of Rio de Janeiro (UFRJ), she led the Endocrine Physiology Laboratory, which now proudly bears her name. At the laboratory, Doris trained and inspired countless students and researchers. Nationally recognized for her exceptional career, she became only the second woman named an Honorary Member of the Brazilian National Academy of Medicine in 2015.

She supervised 27 master’s and 13 doctoral students. She published seminal papers in thyroid research and authored 28 book chapters. Her legacy lives on in the generations of physicians, scientists, and educators she trained, inspired, and guided. The list of students she mentored is extraordinary and includes some of the most important leaders in Brazilian endocrinology, such as Egberto Moura, Carmen Pazos de Moura, Denise Carvalho, Belkiss Cardoso, Hans Graf, César Luiz Boguszewski, Gisah Amaral, Tânia Furlanetto, Yara Medeiros, Milena Braga, Marisa Breitenbach, Tamar Frankenfeld, Vânia Costa, Celly Nascimento, Débora Moreira, Michelle Marassi, Monique Monteaux, and Denise Boechat. Many of them were mentored by Doris as undergraduate, master’s, and Ph.D. students. As a mentor, she treated every student with fairness, respect, and the utmost seriousness. She taught by example—not only through her scientific rigor but also through her integrity, generosity, and unwavering commitment to education.

A founding member of the Brazilian Society of Physiology, she also served as vice-president and president of the Latin American Thyroid Society. She was an active member of the Brazilian Society of Endocrinology and Metabolism (SBEM), particularly within its Basic Endocrinology and Thyroid Departments—both of which she helped establish and in which she served as vice-director, director, and board member.

**Figure 5.**
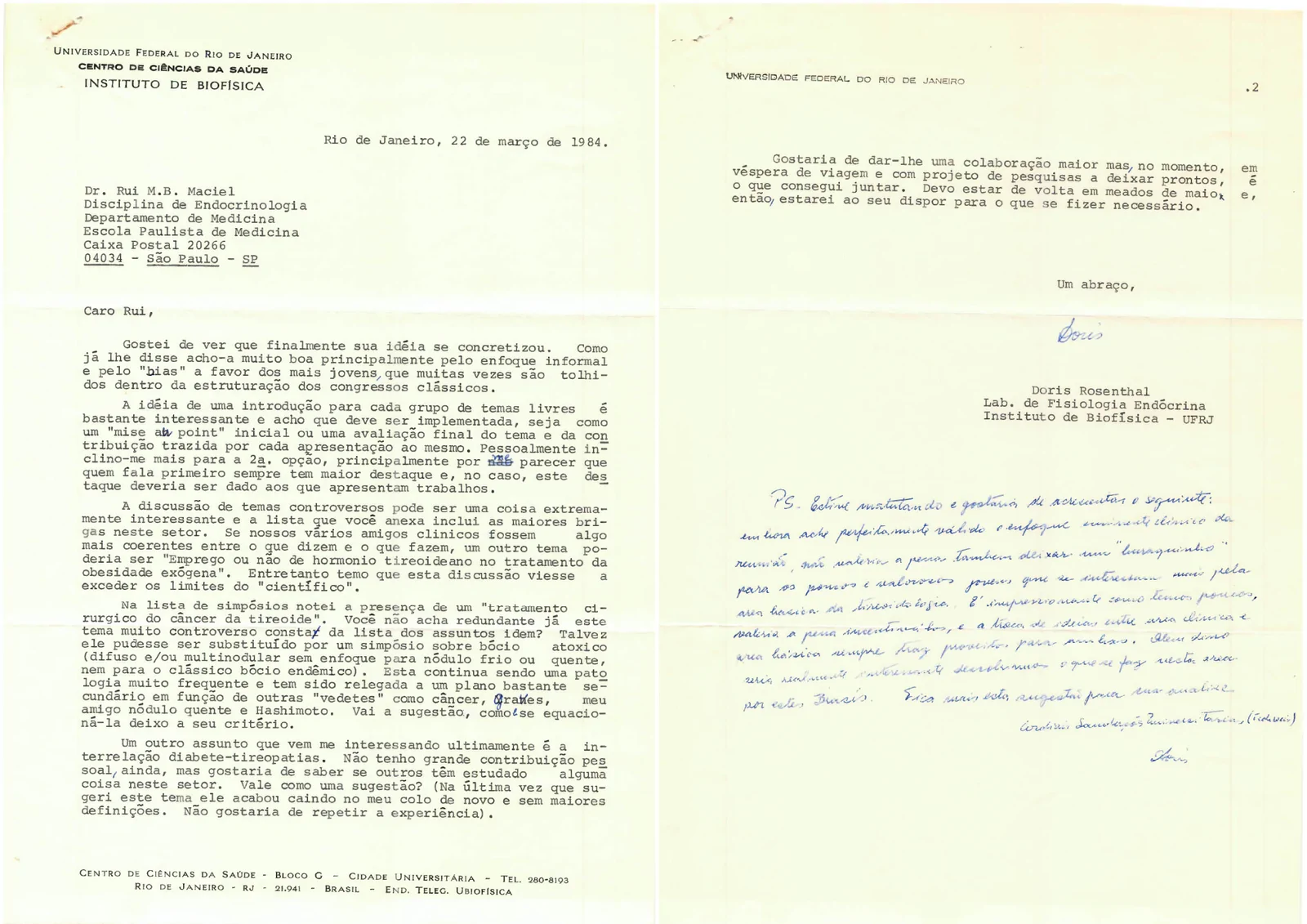
Reply to the invitation from Rui Maciel to create the Brazilian Thyroid Meeting, 1984.

Among the many legacies she leaves behind is the creation of the Brazilian Thyroid Meetings (*Encontros Brasileiros de Tireoide* — EBT) with João Romaldini and Rui Maciel. On March 22, 1984, as plans for the first edition of the event were underway, she wrote to Rui Maciel (**Figure 5**). Her words already revealed the special care and attention she devoted to young physicians, besides the integration between clinicians and basic scientists:

*“Dear Rui,*

*I was delighted to see that your idea has finally become a reality. As I have told you before, I think it is an excellent idea, particularly because of its informal approach and its emphasis on younger participants, who are so often constrained by the structure of traditional conferences.*

*The idea of an introduction for each group of free-paper presentations is very interesting and, in my opinion, should be implemented—either as an opening overview or as a concluding assessment of the subject and the contributions made by each presentation. Personally, I am more inclined toward the second option, mainly because the person who speaks first always receives greater prominence, and in this case that prominence should belong to those presenting their work.”*

Her international recognition included membership in the American Thyroid Association and the European Thyroid Association. Throughout her career, she received numerous honors, including the Latin American Thyroid Award, the inaugural “Encontro Brasileiro de Tiroide” Lifetime Award, the José Schermann Award from IEDE, the SBEM-PR Award, and the *Prata da Casa* Award from IBCCF/UFRJ. In 2023, she received the UFRJ Minerva Medal for Academic Merit, following approval by the university’s highest governing body—an honor that fittingly recognized a lifetime devoted to the institution and to Brazilian science.

Professor Doris Rosenthal was part of a generation of faculty who became pillars of higher education in Brazil. Her commitment to science, the creation of knowledge, and the training of young researchers helped establish the full-time academic career model, which proved essential to the country’s scientific and technological development.

After her mandatory retirement in 2002, she continued working as a professor and researcher at the Endocrine Physiology Laboratory of the Carlos Chagas Filho Institute of Biophysics at UFRJ. More than a decade ago, the laboratory was named in her honor, following the Institute Council’s unanimous approval.

Doris remained active as a collaborating professor and volunteer. Although the COVID-19 pandemic inevitably curtailed her activities, she continued to follow developments in her field and remained intellectually engaged until the very end of her life.

We now bid farewell to an exemplary Brazilian scientist and a remarkable human being. She trained a legion of professionals who continue to share knowledge and uphold the highest standards of excellence across Brazil and abroad. Through them and the institutions she helped shape, her influence will endure.

Doris Rosenthal leaves far more than an extraordinary academic record. She leaves a living legacy of courage, knowledge, perseverance, and devotion—a life dedicated to science and teaching in every sense.
